# Modalities for teaching responsible and ethical conduct of research online: Lessons learned from an undergraduate workshop in Utah

**DOI:** 10.1371/journal.pone.0296461

**Published:** 2024-02-07

**Authors:** Jesse L. Morris, Erin Trouth Hofmann, Weihong Wang, Michael Ault, Sylvia Bradshaw, Trent Foxley, Patrick Thomas, Caren J. Frost

**Affiliations:** 1 Department of Geography, University of Utah, Salt Lake City, Utah, United States of America; 2 Department of Sociology and Anthropology, Utah State University, Logan, Utah, United States of America; 3 Department of Earth Science, Utah Valley University, Orem, Utah, United States of America; 4 Department of Communication, Weber State University, Ogden, Utah, United States of America; 5 Office of Sponsored Programs, Agreements, Research, and Contracts Office, Southern Utah University, Cedar City, Utah, United States of America; 6 Office of Foreign Influence, University of Utah, Salt Lake City, Utah, United States of America; 7 Office of General Counsel, Weber State University, Ogden, Utah, United States of America; 8 College of Social Work, University of Utah, Salt Lake City, Utah, United States of America; Lamar University, UNITED STATES

## Abstract

The COVID-19 pandemic disrupted scientific research, teaching, and learning in higher education and forced many institutions to explore new modalities in response to the abrupt shift to remote learning. Accordingly, many colleges and universities struggled to provide the training, technology, and best practices to support faculty and students, especially those at historically disadvantaged and underrepresented institutions. In this study we investigate different remote learning modalities to improve and enhance research education training for faculty and students. We specifically focus on Responsible and Ethical Conduct of Research (RECR) and research mentoring content to help address the newly established requirements of the National Science Foundation for investigators. To address this need we conducted a workshop to determine the effectiveness of three common research education modalities: Live Lecture, Podcast, and Reading. The Live Lecture sessions provided the most evidence of learning based on the comparison between pre- and post-test results, whereas the Podcast format was well received but produced a slight (and non-significant) decline in scores between the pre- and post-tests. The Reading format showed no significant improvement in learning. The results of our workshop illuminate the effectiveness and obstacles associated with various remote learning modalities, enabling us to pinpoint areas that require additional refinement and effort, including the addition of interactive media in Reading materials.

## Introduction

The COVID-19 pandemic highlighted the struggle that higher education institutions face in addressing the remote learning needs of undergraduates, especially at institutions that mainly serve disadvantaged and underrepresented students [1–4]. As noted by Hermosisima et al., innovative technological opportunities mean that institutions of higher education need to collaborate for “equitable access to reliable internet connections, support disadvantaged learners, and foster creativity through interactivities” [5]. In addition, new measures of how well learning objectives are met are crucial to appropriately situation technology to augment teaching and learning landscapes. With the advent of recent technologies, the potential for conducting activities related to questionable research practices and research misconduct is rising—providing learning options for undergraduates to better understand the ethical parameters of research is key in training the next generation of responsible researchers [6].

In the Intermountain West region of the United States, student learners from rural, Indigenous, and Hispanic/Latinx communities are more likely to attend primarily undergraduate institutions (PUIs) than are students from more advantaged backgrounds [7, 8]. Recruitment of disadvantaged and underrepresented students into STEM research helps students persist to graduation and provides opportunities for future graduate study [9]. Yet, many PUIs lack resources to offer specialized training in responsible and ethical conduct of research (RECR) and Research Mentoring for STEM students and faculty, which are critically important for sustaining a successful research program. The COVID-19 pandemic highlighted just how many elements of online learning still needed to be addressed such that online education is appropriately provided in the classroom of the future [3]. Plagiarism, one of the federally defined components of research misconduct, is one area wherein the access to technology and artificial intelligence without specific parameters for understanding their ethical use is of critical concern [3, 10]. Thus, there is a pressing need to engage PUIs in RECR and research mentoring trainings to enhance student learning, research quality, and student success. The urgency and importance of this need is underscored by new requirements from the National Science Foundation (NSF) as outlined in the current Proposal and Award Policies and Procedures Guide (PAPPG) [11]. Specifically, Chapter II.D.1.d requires that organizations seeking NSF funding certify that investigators and senior personnel receive RECR and research mentoring training for proposals submitted after July 31, 2023. As a result of the NSF policy changes, many colleges and universities, especially PUIs, Historically Black Colleges and Universities (HBCU), and Tribal Colleges, may face challenges in responding to these proposed changes in a timely manner [12]. In the near term, these challenges may hinder institutions with low research education capacity from competing for NSF funding, ultimately defying the critical component of broader impact from NSF research. Additionally, faculty at these institutions often lack training opportunities to engage in effective mentoring relationships, which could ultimately limit the retention and success of these students in STEM research fields.

The COVID-19 pandemic highlighted the need for changes in classroom instruction; however, at the current time there is a lack of curriculum guidance on what works best in the classroom in terms of content on research ethics and responsible conduct of research [13]. As Rong Goh et al. noted, “teleconferencing-based platforms” can be used for teaching; however, the impact of their use is still being evaluated [13]. In addition, Peters and Stamp support the idea that technological opportunities in the 21st century offer a variety of new ways to present materials to students [14]. For example, the use of “embedded simulations” that provide experiential learning for students appears to be particularly impactful [14]. This concept is especially true when learners are “immersed” in the simulation environment [14]. Moloney et al. describe the effect of “simulation-based education” as a method to “enhance learning experiences for students” [15]. Their study made use of online surveys to evaluate the outcomes of this type of learning with students reporting that simulation-based learning was particularly informative and useful for reflecting on the materials to developing critical thinking skills necessary for their professional work [15]. Finally, Karkarougkas and Abdellatif explored the use of active learning, which has been described in the literature for over 20 years, including the successful use of the flipped classroom modality [16]. This model provides information outside the classroom so that the “learning” occurs in the classroom setting through discussions and problem-solving activities allowing students to be more than “inert learners” [16, 17]. Work outside the classroom may include watching videos, completing readings, and/or listening to podcasts such that the learner has reviewed these items and can bring their new knowledge and questions to the classroom setting [16].

As Patel et al. examined, use of case studies and discussions about the information being taught promotes a “better understanding of ethical practices and its [sic] importance in conducting research” [18]. Structuring class sessions based on focused module-by-module layout, such that one module was “small group case-based training” and one module was “theme lectures,” was effective in ensuring that learning objectives were achieved and survey data indicated that along with group discussions this type of learning was particularly effective [18]. Research education is fundamental to establishing RECR and research mentoring best practices at institutions of higher learning and is relevant to nearly every discipline supported by NSF—especially STEM research fields [19, 20]. Institutions with the highest research productivity (e.g., R1s) allocate resources and personnel to develop and provide research education content. In response to the COVID pandemic, many R1 institutions leveraged technology-enhanced instruction to promote accessibility, permanence, and transferability, such as asynchronous content, webinars, and audio podcasts. Since many institutions, particularly PUIs, lack similar research education infrastructure, a natural outcome of this policy is that faculty and students at PUIs will be inadequately prepared to meet the new NSF requirements and these institutions may seek out remote learning opportunities from R1 institutions and other groups to help bridge the infrastructure gap.

Given the need to provide educational opportunities using various modalities, due to the COVID-19 pandemic and potential upcoming pandemics, this study sought to determine success in learning outcomes using three different teaching modalities: two dynamic modalities (discussions and podcasts with discussion) and one status modality (reading only). For this study we used traditional learning (readings) and more active learning (podcasts and discussions) to determine if the information consumed during a class setting about the data lifecycle would be comparable for knowledge gain. The goal of this study was to determine which remote learning modalities are best suited to broaden access to research education training across institutional designations by using five public universities in Utah as a pilot study.

## Background

The existing literature on research ethics (i.e., RECR) training highlights the challenges of implementing effective education programs and the inadequacy of many current approaches for research ethics content. Across STEM fields, as well as in the corporate sector, most studies show that the impact of ethics training programs are modest at best [21–24], although recent reviews suggest that such programs are improving [19]. The challenges to both developing and implementing ethics training for STEM faculty and researchers are many, but the literature highlights four key focal areas as summarized below.

First, identifying best practices in RECR training is challenging due to a lack of consistency and transparency in published research evaluating ethics education programs. The goals and approaches of ethics training are diverse, and even within disciplines there are not standardized definitions of training approaches. As a result, programs with different goals might be described with similar terms, while programs with largely similar goals could be described in quite different terminology [25]. Similarly, there are many ways to measure learning outcomes [20, 25]. Meta analyses of the effectiveness of different approaches to ethics education have been limited by the failure of many published studies to include essential methodological information [24]. Accordingly, to achieve innovations in research ethics teaching, rigorous assessment and evaluation of training programs must be conducted.

Second, much existing research ethics training (as well as ethics in other fields) focuses on micro-ethics, specifically ethical interactions with one’s colleagues and collaborators, and on regulatory compliance [26, 27]. In STEM research, ethics regulations are both complex and changing. While regulatory compliance is necessary, education that focuses only on compliance will soon become outdated as regulations evolve. The Committee on Federal Research Regulations and Reporting Requirements emphasized the importance of fostering “a culture of integrity among academic leaders, faculty, postdoctoral trainees, students, and staff, and institutional administrators” [28]. Doing so requires research training that focuses on developing ethical reasoning while focusing on macro-level ethical questions.

Third, training in research ethics needs to be both ongoing and interactive, particularly when the goal is to develop competency in ethical reasoning [27]. Some researchers on this topic argue that online ethics training is inherently inferior to in-person training, because in-person training allows for shared reflection and dialogue, as well as facilitates long-term relationships that are necessary for ongoing training [24, 27]. However, online learning is an essential part of today’s university landscape and eschewing online training opportunities disadvantages and/or excludes students and faculty at tribal and rural institutions, and at those institutions that historically serve first-generation and non-traditional students, such as PUIs. These institutions already face the greatest struggles in responding to the complex and changing regulatory framework in STEM research [28], including recent changes at NSF. The challenge, therefore, is to design interactive online training that encourages reflection and focuses on how researchers can be ethical in their work rather than emphasizing ineffective practices and prevention alone.

Finally, training in ethics is ineffectual when trainees perceive that the content is irrelevant to the “real world” of their discipline or profession. For example, a study focusing on engineering students found that commitment to ethical principles and socially conscious engineering declined after exposure to seminars in engineering ethics, even at apparently well-designed programs [21]. Therefore, research ethics education should not be limited to specific ethics trainings, rather it should be incorporated in entire curricula and disciplinary cultures, and in university settings should target students, faculty, and staff.

## Methods

### Overview

On September 14, 2022, we delivered an online workshop over Zoom titled “Broadening Impact: Data Basics for the Undergraduate Researcher” to determine which education modalities are most effective in delivering research education trainings to STEM undergraduates. We organized a workshop for undergraduate students in Fall 2022 that aimed to investigate three common virtual education modalities: Live Lecture, Audio Podcasts, and Reading. We selected two thematic foci that provide the foundation to support RECR, specifically (1) Managing Privacy and Anonymity and (2) Ensuring Secure Data Storage. Consent cover letters were provided to all potential participants and the letters were reviewed at the beginning of the workshop so that any questions could be addressed. Student participants were asked to take pre- and post-tests so that we could determine change in knowledge as a result of the workshop. The pre- and post-test questions were administered online through Canvas, immediately before and immediately after the workshop. Faculty and student participants were randomly assigned to one of the three educational modalities described above.

**Fig 1:**
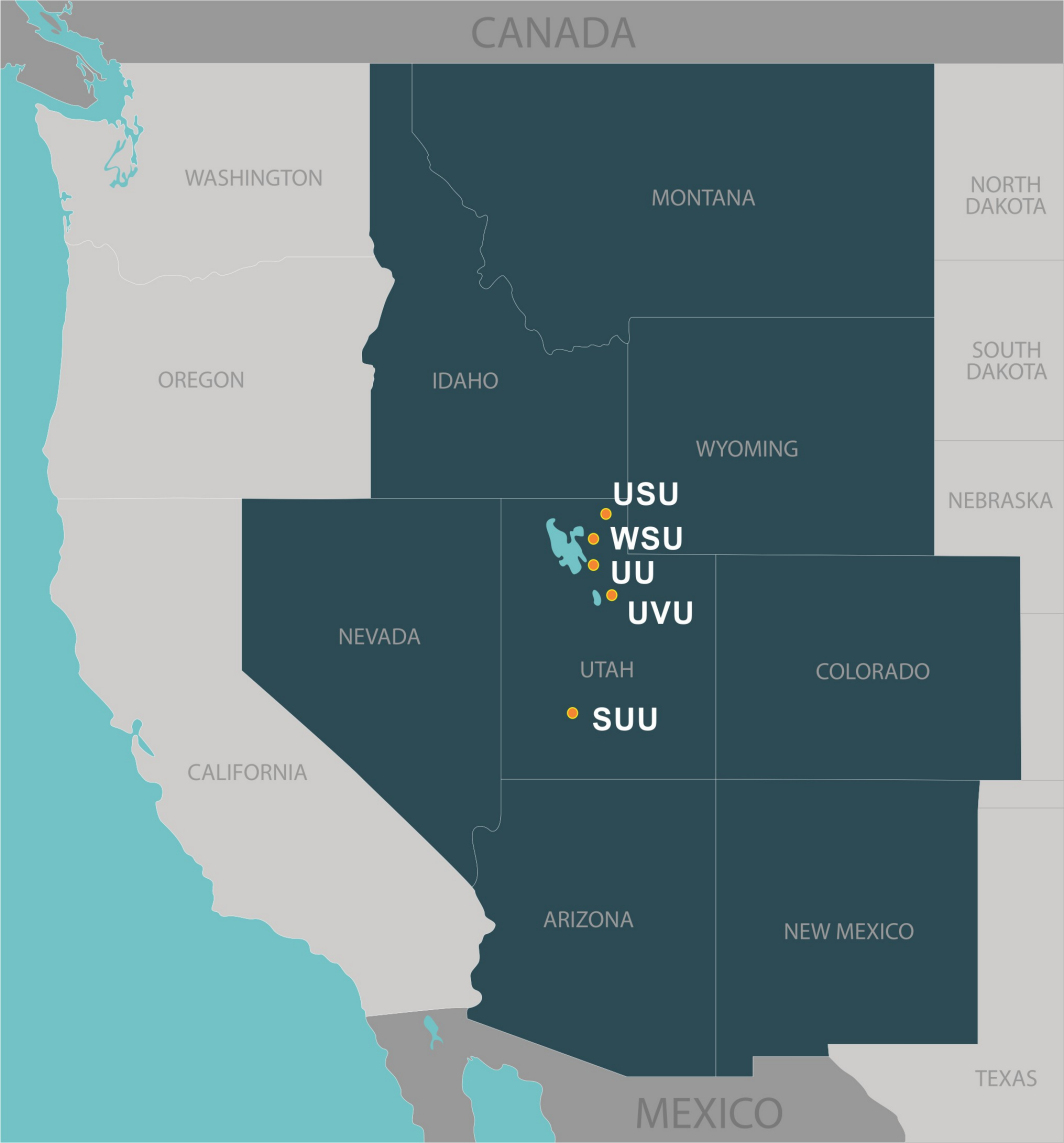
Map depicting the states of the Intermountain West region in dark blue shading with the locations of the IDERC institutions highlighted.

Faculty and student participants were recruited across five public higher education institutions in Utah: Southern Utah University, University of Utah, Utah State University, Utah Valley University, and Weber State University (Fig 1). Participation was incentivized by offering $25 Amazon gift cards for those who completed the pre- and post-test questions and stayed for the 90-minute duration of the workshop. The project team also recruited an Advisory Panel of 9 faculty and staff (5 female, 4 male) representing all five institutions representing several STEM (e.g., biology, chemistry) and social science disciplines (e.g., psychology, ethnic studies). The Advisory Panel consisted of five assistant professors, one associate professor, two professors, and one dean. The Advisory Panel were split evenly among the three modality groups and observed the workshop and provided feedback to the project team at the conclusion of the event. Each Advisory Panel member was compensated with a $100 Amazon gift card.

**Fig 2:**
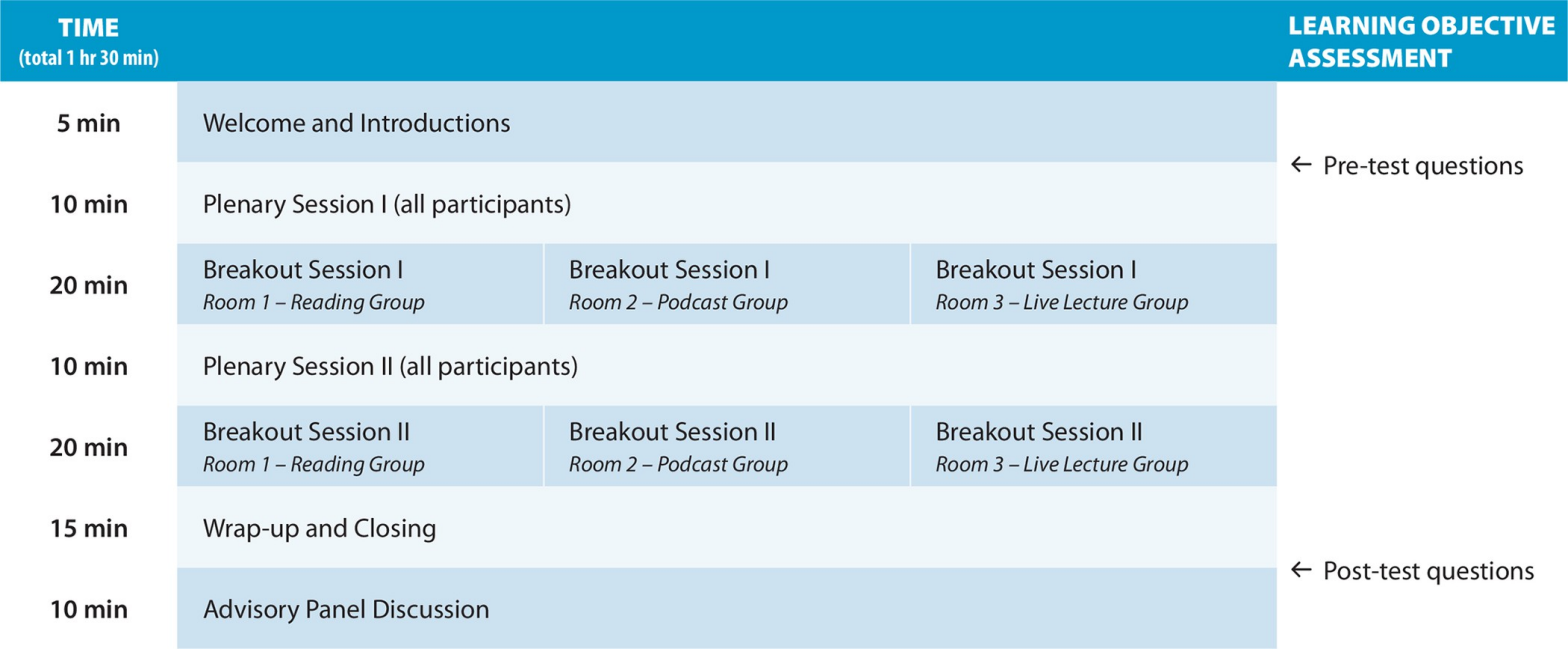
Overview of preliminary workshop organization. Learning outcomes assessed in an administered as pre- and post-test questions (righthand side of panel) are summarized in Tables 1 and 2 below.

The workshop participants were divided into three breakout groups around an education modality for the RERC training: (1) Live Lecture Group; (2) Podcast Group; and (3) Reading Group (Fig 2). The Live Lecture Group attended a breakout room where facilitators provided lecture-based content on data management. The Podcast Group attended a breakout session where they listened to pre-recorded audio podcasts developed by the University of Utah Office of Research Education that featured a scripted conversation and narrative. The Reading Group attended a breakout session where information was provided in text only. The Reading Group reviewed case studies pertaining to data confidentiality and secure data storage. The data confidentiality content featured excerpts from data confidentiality agreements documents gathered from the participating institutions. The secure data storage document centered on a news article describing the preventable aspects of a recent high-profile data breach in Canada. University of Utah Institutional Review Board (IRB) approval was granted prior to execution of the workshop (IRB 00154720). In the sections below, we discuss the evaluation methods applied, the results of the evaluation, and lessons learned for future research training activities.

### Workshop evaluation methods

We evaluated the workshop using both qualitative and quantitative methods. Undergraduate workshop participants were asked to complete both a pre- and post-test during the workshop. To create the pre- and post-tests, we first developed a set of five learning objectives (LO) outlined in Table 1. LO2 and LO3 were covered during the first plenary and breakout sessions, while LO4 and LO5 were covered in the second plenary and breakout sessions. LO1 refers to topics that were covered across the entire workshop.

**Table 1:** Summary of learning objectives (LO) and question banks.

	Learning Objective (LO)	Total in Question Bank	Each Student Answers
LO1	Understand the importance of data management in human subjects research.	5	2
LO2	Understand the concept of the data lifecycle, and name and describe its stages	5	2
LO3	Identify the key ways in which issues of data management will affect research at the planning stage, during data collection, and after data collection is complete.	9	3
LO4	Define and understand the concept of confidentiality in human subjects research.	8	3
LO5	Incorporate information on confidentiality into an informed consent document.	4	2

For each LO, we developed a bank of 4–9 questions, which were used for both the pre- and post-tests. The questions were a mix of true/false and multiple choice. The tests were administered to participants using the Canvas learning management system. Canvas is an educational management platform available at all five institutions of higher education. For each participant test, Canvas randomly selected two or three questions for a student to answer. LO1, LO2 and LO5 were given two questions each, and LO3 and LO5 got three questions each, for a total of 12 questions per student, per test. In addition to the quantitative evaluation, we conducted a focus group after the workshop with the Advisory Panel, who were asked to identify areas of both strength and weakness of the workshop.

## Results

A total of 88 undergraduate students participated in the workshop. The pre- and post-test results presented in Table 2 capture only those participants who: 1) completed both the pre- and the post-test, and 2) joined both the first and second breakout sessions. These two restrictions reduced the number of students in the analytical sample to 44, including 15 in the Live Lecture Group, 12 in the Podcast Group, and 17 in the Reading Group.

**Table 2:** Mean number of correct answers in the pre- and post-tests, by learning objective (LO), and breakout group.

	Live Lecture Group (n = 15)		Podcast Group (n = 12)		Reading Group (n = 17)	
	*Pre*	*Post*	*Pre*	*Post*	*Pre*	*Post*
LO1	1.13	1.20	1.08	1.00	1.06	1.00
LO2	1.20	1.53	1.42	1.16	1.17*	1.53*
LO3	1.27*	1.80*	1.58	1.67	1.47	1.41
LO4	1.47*	2.00*	1.5	1.17	1.35	1.65
LO5	1.40	1.00	1.41	1.33	1.17	1.24
Total	7.47*	8.53*	8.00	7.33	7.24	7.82

*Mean score on the post-test is significantly higher than mean score on the pre-test, p < .1 (one-tailed test)

As shown in Table 2, the difference between pre- and post-test scores is not large. The Live Lecture Group had a one-question improvement in scores, going from an average of ~7.5 to an average of ~8.5 questions correct (out of 12 total questions). The Reading Group had a smaller (and non-significant) improvement in total score, while the Podcast Group saw a decline in scores (although not a significant decline). Improvements were concentrated in LO2, LO3, and LO4, demonstrating that learning occurred across both breakout sessions, although not in every area.

As a robustness check, we considered the possibility that the results were driven by a few poorly written questions. The quiz tool in Canvas creates a “discrimination index” metric for each question. The discrimination index is the point-biserial correlation between students’ overall quiz score and their probability of getting the correct answer on individual questions. Correlation coefficients of .24 and below are flagged by Canvas as indicating potentially problematic questions [29]. In the post-test, two questions were included from the LO1 question bank. In the post-test, two questions from the LO1 question bank, one question from LO2, two questions from LO3, one question from LO4, and one question from LO5 were flagged. We created an alternative score that did not include these seven questions. Without the flagged questions, there were fewer instances where the mean score dropped between the pre- and post-tests, but the overall patterns remained broadly similar, indicating that the patterns described above were not influenced by a few problematic questions. An interitem correlation was also performed for each LO. Alpha values range from .62 - .79 across as LOs, while alpha values ranged between .62-.85 without LO2. LO2 had a poor interitem correlation (.25) and likely because LO2 had few questions and therefore the LO2 results may not be meaningful.

### Advisory panel recommendations

The strong evidence of learning among the Live Lecture Group aligned with the observations of our Advisory Panel, who generally found the Live Lecture Group sessions to be productive. The Advisory Panel found that the Reading Group was not engaging to students, and did not provide enough direction, leading to confusion. The Podcast Group, however, was viewed as more engaging. Another concern of the Advisory Panel is that the workshop tried to cover too many LOs in a short timeframe. Using one case study per topic for two different topics was likely too much information and sticking to one topic for a 90-minute workshop may have been a more effective approach. Finally, although the Advisory Panel was not originally intended to complete the pre- and post-tests, a few did so. One faculty participant reported in the focus group that she found one of the questions confusing, and to this individual none of the provided answers appeared to be correct.

## Discussion and conclusions

Our preliminary results from the pre- and post-surveys revealed that the Live Lecture Group demonstrated the greatest learning gains, while differences between pre- and post-test scores in the Podcast and Reading groups were not statistically significant. This evaluation has several important implications for the development of future workshops and development of training materials. The Advisory Panel provided four main themes to improve the workshop impacts content. First, the Advisory Panel suggested that future workshops be reduced in scale and participants be allotted more time with to engage with workshop materials. Second, we should focus on elements of single educational modality (i.e., podcasts). Third, the Advisory Panel suggested that the materials in the Reading Group could be enhanced by including interactive media and/or video recordings of key materials. The Advisory Panel recommendations and our plan to incorporate their suggestions are discussed in greater detail below. Fourth, the Panel recommended that we provide a registration link and require students to complete any pre-test or other pre-workshop surveys prior to the workshop to save time and maintain a seamless training process.

While the Live Group sessions provided the most evidence of learning and were well received by the Advisory Panel, live sessions are not always feasible nor are they necessarily accessible to all students who need training in RECR. The podcast format was viewed positively but produced a slight (and non-significant) decline in scores between the pre-and post-tests, indicating that some students may have been confused by the podcast and retained incorrect information. We will pair future podcasts with complementary information in writing or another format and also add a summary (or recap) and the conclusion of the podcast to reiterate the takeaway message(s) [30]. When materials are presented in text format, they should be paired with interactive media, including videos and graphics, to better engage students.

In organizing future online workshops to test learning modalities, we will devote more time to developing to ensure internal consistency. We will also provide a workshop registration link and require students to complete any pre-test or other pre-workshop surveys beforehand. This requirement may increase students’ commitment to the workshop and will save time at the beginning and allow late arrivals to still participate. As mentioned above, allowing for substantially more time/information per learning objective is essential. Given the poor performance on the post-test, and the concern expressed about the questions by one Advisory Panel member, we will devote more attention to our evaluation strategy in the future. We will also refine the pre- and post-test questions to ensure that they are (more) closely linked with the LOs to support improved assessment of the LOs. Additionally, we will integrate the development of the evaluation metrics and the workshop content. Furthermore, we will request input on evaluation metrics from researchers and compliance professionals outside of the project team, and consult with campus offices to expand participation and enhance accessibility.
